# Effect of the sequence data deluge on the performance of methods for detecting protein functional residues

**DOI:** 10.1186/s12859-018-2084-7

**Published:** 2018-02-27

**Authors:** Diego Garrido-Martín, Florencio Pazos

**Affiliations:** 1 Computational Systems Biology Group, Systems Biology Program, National Centre for Biotechnology (CNB-CSIC), c/ Darwin, 3, 28049 Madrid, Spain; 2 Present address: Centre for Genomic Regulation (CRG), The Barcelona Institute for Science and Technology, c/ Dr. Aiguader, 88, 08003 Barcelona, Spain; 3 Present address: Universitat Pompeu Fabra (UPF), Plaça de la Mercè, 10-12, 08002 Barcelona, Spain

**Keywords:** Sequence database, Functional residue, Conserved positions, Family-dependent conserved position, Specificity-determining position, Functional subfamily

## Abstract

**Background:**

The exponential accumulation of new sequences in public databases is expected to improve the performance of all the approaches for predicting protein structural and functional features. Nevertheless, this was never assessed or quantified for some widely used methodologies, such as those aimed at detecting functional sites and functional subfamilies in protein multiple sequence alignments. Using raw protein sequences as only input, these approaches can detect fully conserved positions, as well as those with a family-dependent conservation pattern. Both types of residues are routinely used as predictors of functional sites and, consequently, understanding how the sequence content of the databases affects them is relevant and timely.

**Results:**

In this work we evaluate how the growth and change with time in the content of sequence databases affect five sequence-based approaches for detecting functional sites and subfamilies. We do that by recreating historical versions of the multiple sequence alignments that would have been obtained in the past based on the database contents at different time points, covering a period of 20 years. Applying the methods to these historical alignments allows quantifying the temporal variation in their performance. Our results show that the number of families to which these methods can be applied sharply increases with time, while their ability to detect potentially functional residues remains almost constant.

**Conclusions:**

These results are informative for the methods’ developers and final users, and may have implications in the design of new sequencing initiatives.

## Background

The pace at which new amino-acid sequences of proteins are deposited in public databases is exponentially growing. Today it is relatively straightforward and inexpensive to sequence an entire genome, whose translation would yield the sequences of the encoded proteins. Recent improvements in sequencing technologies are boosting this trend [1], so that not only genomes of representative taxa are available, but also of similar strains or even individuals.

One way of taking advantage of this deluge of sequence information is to use sequence comparison methods to retrieve and compare similar proteins. Protein multiple sequence alignments (MSAs) have been long used to obtain functional and structural information [2]. In a MSA, homologous proteins (i.e. those sharing a common ancestor) are represented in such a way that evolutionarily equivalent residues stack together (in the same column in the most common representation). These alignments allow not only to perform global structural and functional inferences (i.e. transferring structure and function between homologous proteins), but also to study finer details at the residue level. Since a column in a MSA (termed “position” in this context) can be regarded as a representation of the amino-acids allowed by the evolution at a particular site in the set of homologs, a lot of information can be obtained studying its patterns of amino-acid change/conservation [3, 4]. Full conservation was the first and most straightforward pattern extracted from MSAs [5, 6]: positions where evolution did not allow any change should have some functional or structural importance. Another informative pattern involves pairs of positions, where the pattern of inter-homolog residue change in one position is not independent but related to that of another, so that the two positions are co-varying. These correlated change patterns are in many cases due to compensatory mutations between residues close in the three-dimensional structure of the proteins, and hence their main utility is to predict residue contacts from sequence information [7–9].

Another mutational pattern indicative of functional relevance is the so-called *family-dependent conservation*. When we can divide the set of homologous proteins into subfamilies according to some functional criteria (e.g. groups with different functional specificities within the global function shared by all the homologs), some positions appear as differentially conserved within these subfamilies. For instance, they are conserved in one group but not in others, or they are conserved within each subgroup but with a different amino-acid. These positions point to protein functional sites related to the functional specificities that determine the group separation, and consequently they are generally termed “specificity determining positions” (SDPs) [3, 4, 10]. For example, within a set of homologous enzymes performing the same catalytic activity, we can define subgroups depending on the substrate specificity. Whereas fully conserved positions would map to the active site residues responsible for the catalytic activity, SDPs would point to those involved in binding the different substrates. Besides differential binding, SDPs could also point to other regions related to functional specificity, such as allosteric regulation sites, residues determining protein stability, etc. Consequently, SDPs complement fully conserved positions as predictors of functional sites from sequence information alone.

There are many approaches to detect SDPs from multiple sequence alignments, e.g. [11–17]. For some reviews and comparisons see [3, 4, 10, 18–20]. Most of these approaches are “unsupervised” in the sense that they use the subfamily definition implicit in the distances between the sequences in the MSA. Some of these methods can explicitly report the assignment of the MSA sequences to subfamilies, concomitantly with the associated SDPs. The maturity of these approaches in terms of performance and usability (e.g. through software with graphical interfaces [21]) makes it possible for them to be routinely used by bioinformaticians and experimental biologists, to perform SDP studies in many proteins of biotechnological and biomedical interest. This is generally done in combination with experimental approaches aimed at mutating these positions in the search for a swap of functional specificities (see [22] for examples).

The performance of these approaches depends on the quality of the MSAs that constitute their only input. It has been hypothesized that the exponential growth of the sequence databases would improve that performance, since the methods would work with larger (potentially richer) sources of information. Nevertheless, this point has not been tested exhaustively or quantified so far. In this work we evaluate how the growth and change with time in the content of sequence databases affect the sequence-based methods for detecting functional sites and subfamilies.

## Methods

**Fig. 1:**
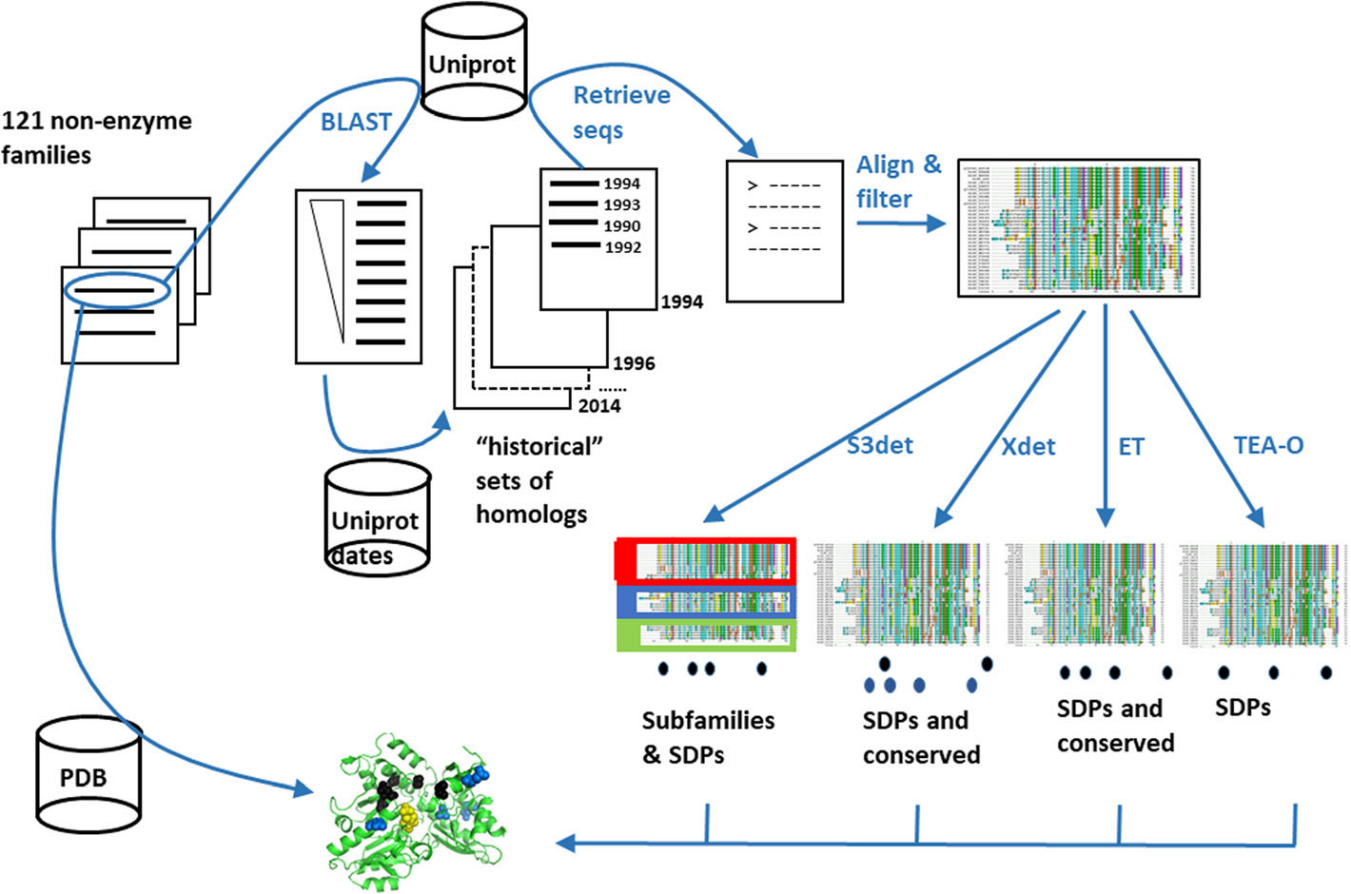
Schema of the methodology. A representative sequence is taken from each of the 121 non-enzymatic families previously used by Chakraborty et al. [10]. Its homologs are retrieved from the current version of Uniprot (top) and yearly subsets of these are constructed based on their publication date. The sequences of these “historical” sets of homologs are retrieved and aligned in an attempt to generate the multiple sequence alignments one would have obtained at a given time point in the past. Finally, the methods for detecting SDPs and functional subfamilies are applied to these historical alignments and the results contrasted with structural information on binding sites if available (bottom)

In order to evaluate how the change in size and content of the sequence databases is affecting the sequence-based methods for detecting functional subfamilies and functional sites, we “recreated” the MSAs that would have been obtained for a test set of proteins at different time points in the past, spanning a period of 20 years. We then applied five methods for detecting functional sites and subfamilies to these “historical” MSAs. In the following, we explain in detail this procedure, illustrated in Fig. 1.

As test set of protein families, we used the “large non-enzyme dataset” previously compiled by Chakraborty et al. [10], which contains 121 families of proteins. This dataset was obtained by automatically filtering an initial set of 7729 families in the PANTHER database [23] by different SDP-related criteria (e.g. enough family members, available functional information, etc.) In order to fit this dataset into our workflow, which aims to generate historical versions of the multiple sequence alignments for a given protein, we did not use the MSAs generated by Chakraborty, but just took the first sequence of each alignment and ran for it the procedure described below, which includes the generation of the alignment.

For each of these 121 sequences, we started by retrieving homologs in the (current) Uniprot database [24] by “blasting” [25] it against this resource (e-value cutoff of 1E-4). We had previously generated a database with the “publication date” of each Uniprot sequence. To simulate the set of homologs that would have been obtained at a given year, from the BLAST output we filtered out the hits with more recent publication dates (Fig. 1). We retrieved the sequences of the remaining hits (publication date equal or earlier than our year of interest) and aligned them with Clustal Omega [26]. A non-redundant version of that alignment was generated by filtering out proteins with more than 95% sequence identity.

We carried out this procedure for the period 1994–2014, in steps of 2 years. Note that this did not yield exactly the same alignments that one would have obtained those years, since the software used (i.e. BLAST and Clustal) have improved and the database content influences the BLAST e-value. Nevertheless, we expect these differences to be minimal, especially given the stringent e-value threshold used (i.e. very close homologs).

We applied to the 11 historical non-redundant alignments of each of the 121 proteins four programs for detecting functional residues and subfamilies: *S3det* [14], *Xdet* [12], *Evolutionary Trace* (ET) [11] and *Two Entropies Analysis Objective* (TEA-O) [27]. S3det is based on a vectorial representation of the proteins in the MSA followed by a dimensionality-reduction step that leads to a multidimensional space where the vectors of similar proteins are close. Subfamilies are detected as clusters in this reduced space. S3det implements a novel procedure for determining the optimal number of axis and protein clusters. An equivalent treatment for the individual positions leads to a “position space” where the vectors of positions with a tendency to be conserved within a given group of proteins cluster in the same region of the space where those proteins are. In this way, S3det concomitantly reports the subfamilies and their associated SDPs.

Xdet is based on the idea that the pattern of residue changes in a SDP position resembles that of the whole family: a group of close sequences (subfamily) would be related to a set of similar (or identical) residues and vice versa. The pattern of changes of a particular position is represented by a matrix with the amino-acid similarities (according to a standard substitution matrix) for all pairs of residues within that position. The pattern of changes for the whole family is represented by an equivalent matrix with the overall sequence similarities for all pairs of proteins. These two matrices are compared with a Spearman nonparametric rank correlation approach so that positions with a high correlation score (≥0.8 in this study) are good candidates to be SDPs. Although Xdet takes into account the subfamily composition of the MSA implicitly (i.e. represented in the whole-sequence distance matrix), it does not report it explicitly. From the Xdet output we also retrieved the fully conserved positions (entropy = 0.0).

ET splits the MSA of the family in different subsets (subfamilies) of different granularity by hierarchically cutting its phylogenetic tree at different levels, from the root to the leaves. The intra-family conserved residues at each level are taken as the predicted functional residues. Since the root of the tree (level 0) generates only 1 subfamily, the functional residues it renders are actually the fully conserved positions, not SDPs. Hence, this approach allows to detect both, fully conserved positions and SDPs. Based on this approach, ET associates a score to each position in the MSA. For this work positions with score 2.0 or better (lower) were selected.

TEA-O follows a similar tree-guided splitting of the family and, for each level, it calculates for all positions their full conservation and an entropy-based parameter aimed at detecting SDPs. A consensus plot is then generated from these level plots, where functional positions cluster in different regions depending on their conservation. Since TEA-O requires an explicit tree as input, we generated it from the corresponding alignment with ClustalW [28] (default parameters). For TEA-O, we took all positions with score 0.3 or better (lower) as the predicted set of SDPs.

Only MSAs with 15 or more sequences were used for retrieving the five sets of predicted functional residues.

For the 121 original proteins, we looked for a homologous structure in PDB using BLAST (e-value ≤1E-3, sequence identity ≥25%, aligned region ≥50%). With these criteria, we found a homolog of known structure for 67 out of the 121 proteins. We retrieved the binding sites annotated for these structures in the FireDB database [29]. For a given set of predicted functional residues in a given family (SDPs and fully conserved sites) we calculated their average distance to the annotated binding sites in the homologous structure, and divided it by the average distances of all residues to the same sites. A value lower than 1.0 would indicate that the set of predicted functional residues tend to be closer than expected to a (potentially) functional region of the protein. While this is not a perfect definition of functional site (i.e. not all the functional sites are close to biding sites and vice versa), it allows evaluating a large enough dataset, especially taking into account that we are interested in relative changes of performance with time, more than the absolute values.

## Results

For a set of 121 protein families, we recreated the multiple sequence alignments (MSAs) that would have been obtained at different time points in the past (1994–2014) in order to evaluate how the change in size and characteristics of the sequence databases affect the MSA-based detection of functional subfamilies and functional sites. See Methods for a detailed description of the procedure.

### The number of available sequences grows exponentially, while redundancy does not

**Fig. 2:**
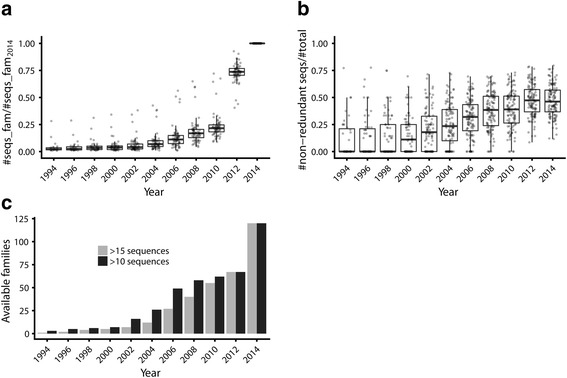
Evolution with time of the sequence repertoire for the families analyzed. **a**) Relative number of sequences available for each family (respect to those available in 2014). The yearly distributions of this parameter for all the families are shown as boxplots. **b**) Same representation for the redundancy within each family (number of sequences in the alignment filtered at 95% sequence identity over those in the non-filtered alignment). **c**) Number of families to which it would have been possible to apply the SDP-based methods considering two thresholds for the minimum number of required sequences

Figure 2A represents the total number of sequences available for the different families each year, relative to those available in 2014. Each boxplot represents the yearly distribution of this ratio for the 121 families. It can be seen that the number of available sequences grows exponentially, in concordance with the overall growth of the whole Uniprot database. If we generate the same plot for the number of sequences in the final MSAs used, that is, after the 95% redundancy removal, the plot is virtually identical (not shown). That indicates that this exponential growth in the number of sequences is not primarily due to redundancy (i.e. that the new sequences added with time are not highly similar to those already available). A better way to quantify this is to actually plot the distribution of redundancies for each family: the ratio of the number of sequences in the final alignment used (95% non-redundant) over the total number of sequences (Fig. 2B). In this case the boxplots show much wider distributions since redundancy varies largely from one family to another, but with a steady increase or even a plateau in the last years (when the increment in the total number of sequences is larger, Fig. 2A).

### The number of workable families grows with time

Figure 2C represents the number of families to which it would have been possible to apply the methods for detecting SDPs and functional subfamilies each year, depending on the minimum number of sequences required in the MSAs (15, used in the rest of this work, and 10). It can be seen that this “coverage” clearly benefits from the accumulation of sequences in databases. For example, in 2004 it would have been possible to apply these methods only to a 10% of the families of our dataset (21% requiring 10 sequences), while 10 years later it was possible to analyze almost all families (120 out of 121).

### The number of detected subfamilies slightly increases but the number of predicted functional sites does not

**Fig. 3:**
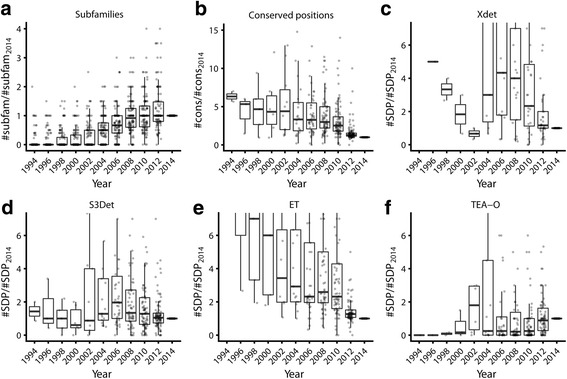
Evolution with time of the number of functional subfamilies and functional sites. **a**) Relative number of subfamilies detected by S3det for each family (respect to those detected in 2014). The yearly distributions of this parameter for all the families are shown as boxplots. **b**) Same representation for the relative number fully conserved positions. **c** to **f**) Same representations for the relative number of SDPs reported by Xdet, S3det, ET and TEA-O respectively

Figure 3A shows the distribution of the number of subfamilies detected by S3det per family and year, relative to the corresponding numbers in 2014. Values larger than 1.0 mean that, for that particular year, S3det detected more subfamilies than in 2014 (in spite of having fewer sequences). It can be seen that, in general, as we accumulate more sequences of members of a family, these led to the definition of new subfamilies. Nevertheless, this increment is very low in the last years and does not recapitulate the exponential growth in the total number of family members (Fig. 2A). A lack of increment in the number of subfamilies in the context of an exponential growth in the total number of family members would indicate that the new sequences are similar to existing ones, hence lying in existing subfamilies. Nevertheless, as commented above, we do not see a large increment in the redundancy, at least with the 95% cutoff we used (Fig. 2B). Consequently, especially in the last decade, it seems that families are enlarged with new members, distant enough not to increase redundancy, but close enough not to define new subfamilies.

Figures 3B to F show the yearly distributions of the number of predicted functional residues (fully conserved positions, and SDPs detected by Xdet, S3det, ET and TEA-O) retrieved from the family MSAs whose characteristics were described above, always relative to these numbers in 2014. In all cases the distributions were fairly broad, since the families are very different in their functional characteristics. Nevertheless, some general trends can be extracted. There is a clear decrease in the number of conserved residues. This is expected because, as we have more sequences, it becomes more difficult for a position to be conserved in all of them. On the contrary, if for instance we only know two sequences for a given family, most positions would be conserved just by chance. Thus, in 1998 we would retrieve on average 4 times more conserved residues from a MSA than in 2014. The patterns for the SDPs detected by the different methods are more complex, but in none of the cases there is an increment in the number of reported positions, except perhaps for TEA-O. For ET there is a clear decrease with time in the number of reported positions. For example, in 2006 we would obtain with this program on average twice as many SDPs as in 2014. Again, these trends are immersed in wide distributions since the behavior of each family in terms of functional subfamilies, associated SDPs, and the functional/evolutionary pressure related to both is very different.

### The accumulation of new sequences does not improve the performance of methods for predicting functional sites significantly

**Fig. 4:**
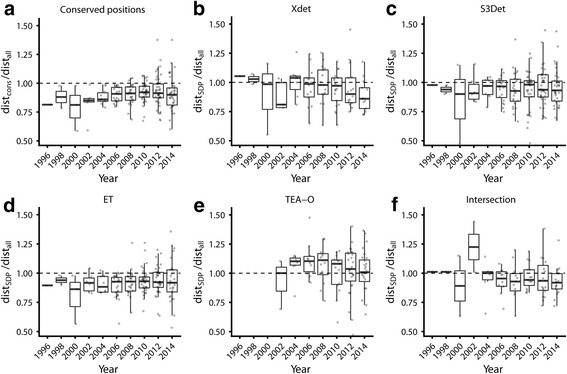
Evolution with time of the relative distances to the annotated binding sites of the detected SDPs and conserved residues. **a**) Average distance of the fully conserved residues to annotated binding sites over the corresponding value for all residues. The yearly distributions of this ratio for all the families are shown as boxplots. **b** to **e**) Same representations for the SDPs reported by Xdet, S3Det, ET and TEA-O. **f**) Same representation for the SDPs reported jointly by Xdet and S3det (intersection between their predictions)

The relative distances from the sets of predicted functional residues to those annotated as “binding” in FireDB is shown in Fig. 4. The first clear observation is that in most cases these values are smaller than 1.0 (see Methods), in agreement with what has been previously reported (e.g. [10, 20]). This indicates that the sets of functional residues (fully conserved, and SDPs reported by Xdet, S3det and ET, as well as the intersection between the first two) are closer to the binding sites than what would be expected by chance. This is not the case for TEA-O, whose predicted sets of positions are farther than expected to the binding sites. Maybe the particular MSAs we are using are not the best for this particular method, it has been optimized to detect other types of functional sites not related to binding (e.g. in surfaces), or it is simply not working in our hands. The best overall performances, as well as the more stables along time, are for fully conserved residues and SDPs reported by ET. As commented in the Introduction, ET also reports fully conserved positions, concomitantly with SDPs.

The most intriguing observation is that in any of the cases the performance increases noticeably as new sequences become available. Even the widely-used conserved positions do not become better predictors of functionality (assessed as closeness to binding sites) as the MSAs become larger. For example, their performance is similar in 2008 and 2014 (Fig. 4A), while the MSAs became 5 times bigger on average (Fig. 2B). Perhaps only the intersection between Xdet and S3det (i.e. taking as predicted functional sites the residues predicted simultaneously by both programs) shows some improvement.

### Example

**Fig. 5:**
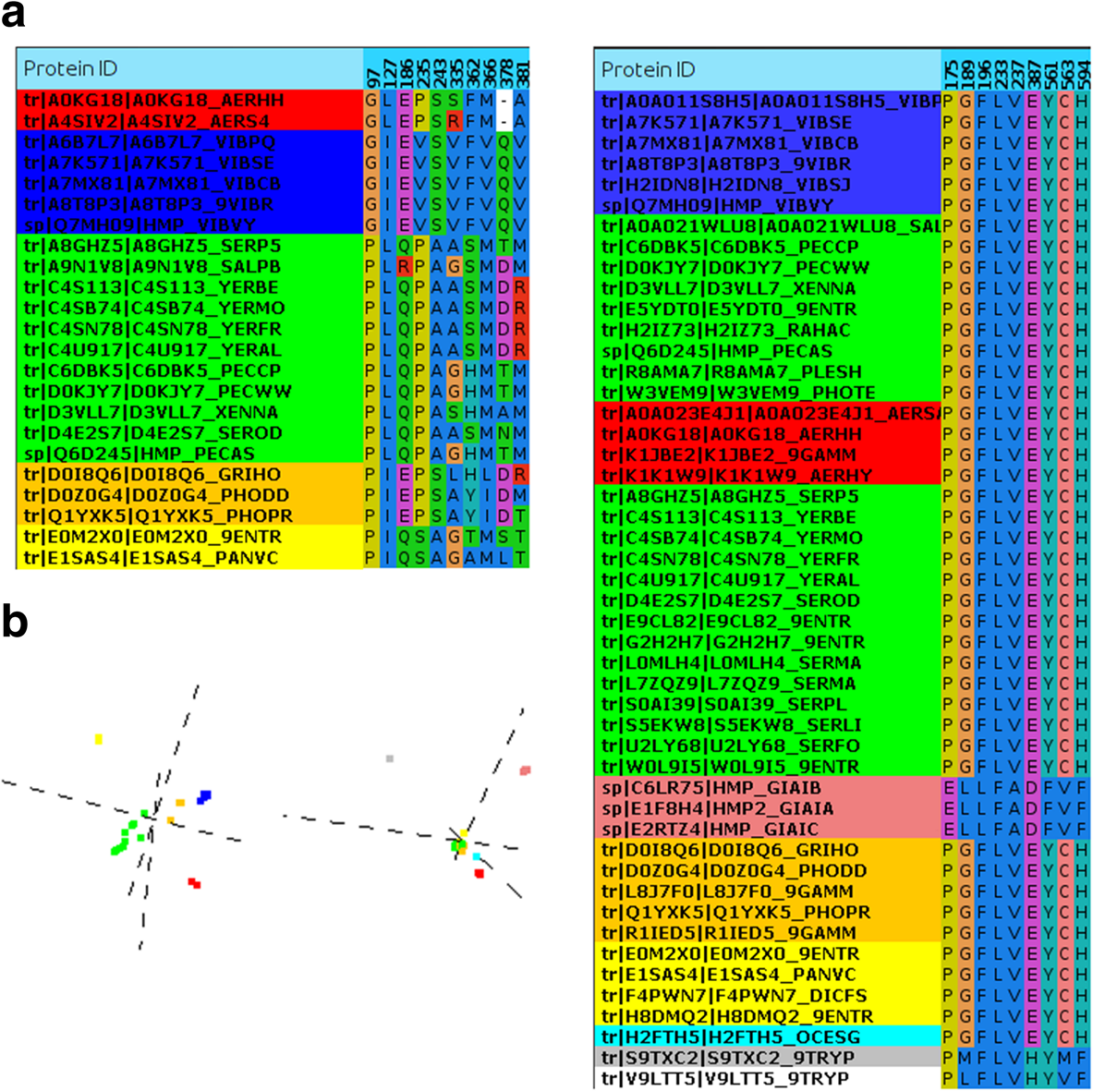
Example of the S3det analysis for a protein family at two time points in the past. The results of S3det for the multiple sequence alignments of leghemoglobin related proteins (PTHR22024) that would have been obtained in 2010 and 2014 are shown. **a**) Detected families and SDPs. **b**) sequence spaces. The representations were generated with JDet [21]

For illustrative purposes, we present here the detailed study of a family of proteins for which the performance of all methods increased with time. For the family of leghemoglobin-related proteins (Panther [23] ID: PTHR22024), the workflow described in Methods generated a final (filtered) MSA with 23 sequences in its historical version of 2010, and 48 sequences in 2014. Figure 5A shows the subfamilies detected by S3det in both alignments. Since most proteins found in 2010 are also present in the 2014 alignment (not all due to the redundancy removal procedure) it is possible to map the subfamilies between both years (depicted in the same color in Fig. 5). Note that the green family split into two due to the addition of new sequences (forced to be depicted in the same color in the 2014 panel for clarity). The new proteins sequenced between 2010 and 2014 also cause the appearance of a new subfamily (pink in 2014). This new subfamily is far from the others in the sequence space, as shown in the vectorial representation generated by S3det (Fig. 5B) (Compare with the more “homogeneous” distributions of the 2010 subfamilies.) In a typical phylogenetic tree this would be a clade far apart from the others.

The MSA columns corresponding to the SDPs reported by S3det are also shown in Fig. 5. The appearance of that very divergent subfamily (pink in 2014) changes completely the pattern of SDPs, since S3det, in the search for positional patterns better reflecting the distribution of the families in the sequence space, will report positions differentially conserved in the new (pink) subfamily versus all the others together (since they are close). This illustrates how the “discovery” of a new subfamily can drastically change the sequence space “landscape” of a given family and, consequently, its associated SDPs. In this particular case, the new SDPs arisen due to the appearance of the new subfamily are better in terms of closeness to the binding sites. Moreover, in 2010 only 2 out of the 10 SDPs identified map in the available 3D structure, which does not cover the whole sequence.

## Discussion

A deluge of sequence data is the most obvious outcome of the modern techniques for the fast and inexpensive sequencing of complete genomes. Consequently, we expect an incremental usage of the methods for predicting protein structural and functional features from these massive sequence data. For this reason, it is important to quantify the effect of the sequence accumulation on these methodologies. In principle, their performance is expected to improve with time, but issues like redundancy could have an opposite effect. Indeed, some studies have questioned the utility of the “blind” unsupervised sequencing of complete genomes in terms of the new information that is actually obtained from them [30, 31]. Other studies have assessed how the temporal accumulation of sequence data affects, for example, the performance of methods for detecting remote homology [32] or the sequence-based prediction of interaction partners [33, 34]. In some cases, the accumulation of sequence data not only improved the methodologies quantitatively but changed them qualitatively, increasing their reliability from a “just above random” stage to the real applicability, side by side with experimental methods. This was the case of the new wave of methods for predicting residue contacts from co-variation information, for which the increase in the size of the MSAs, together with methodological improvements, took them to a point where they can predict the 3D structure of a protein when enough homologs (in the order of hundreds or thousands) are available [7–9]. Methods for predicting SDPs and other functional residues are now part of the standard toolboxes of molecular biologists and are being increasingly used [22]. Current workflows implementing these methods, nowadays do not take into account the composition of the sequence databases, and use all sequences available in these, eventually with a simple redundancy removal. For these reasons an equivalent study on the effect of the sequence accumulation on their performance is timely.

We describe here the first study aimed at getting insight into how the growth of the most widely used protein sequence database affects the performance of the methods for predicting functional subfamilies and functional sites in proteins. As expected, most of the parameters assessed show a broad distribution for the 121 families studied. This is because the families are very different in their functional aspects and so are the evolutionary pressures shaping their sequences. Nevertheless, despite this variability, some global trends could be extracted.

The most obvious general observation is that, as new sequences accumulate, the number of families to which these approaches can be applied drastically increases. However, neither the number of predicted functional sites, nor their accuracy, follow the same trend. Here it is important to take into account that we use a quite stringent criterion to define “functionality”: closeness to annotated binding sites. These are not the only “functional sites” in a protein. However, in this particular work we are interested in the relative change in performance with time, and not in the absolute values. Using a different criterion for functionality could drastically change the performance, but we do not expect it to change its temporal trend. There are specific reviews focused on detailed evaluations of the methods’ performances and their comparison, e.g. [10, 20].

The reason for this intriguing lack of improvement is not clear and, according to our own results, it is not due to the accumulation of redundancy in databases. It might happen that methods are developed in a particular time point and, as a consequence, even if not intended, adapted to a particular database content, reflected in the composition of the dataset used for training/testing. If this is the case, the message to developers would be to try to better assess how the foreseen changes in the MSAs of their datasets would affect the performance (e.g. removing/adding redundancy, simulating the incorporation of new members of the family, …) Getting additional insight into the reasons behind this unexpected lack of improvement would involve following in detail the individual families along time, besides obtaining the global figures we present here. For example, how the sub-family composition changes with time, adding or removing members, how the set of SDPs varies, etc. While this can be easily done for a small set of cases, (we actually show an example for a particular family), quantifying it for a large number of families is still challenging.

## Conclusions

Understanding how the size and composition of the sequence databases affect the performance of the approaches for detecting functional subfamilies and functional residues would allow not only to have a clearer idea about which results to expect, but also to re-direct and better design sequencing efforts, either globally of for particular cases. Our results show that the mere “blind” accumulation of sequences in databases does not help this kind of methodologies. Consequently, other alternatives have to be devised. For example, if the price of sequencing technologies drops enough, we may think of a future where it would be possible to (re)sequence the strains/variants more adequate for the MSA of our family of interest, instead of just relying on the information present in the databases.

## Abbreviations

MSA: multiple sequence alignment
SDP: specificity determining position
